# Statistical Image Properties in Large Subsets of Traditional Art, *Bad Art*, and Abstract Art

**DOI:** 10.3389/fnins.2017.00593

**Published:** 2017-10-25

**Authors:** Christoph Redies, Anselm Brachmann

**Affiliations:** Experimental Aesthetics Group, Institute of Anatomy I, Jena University Hospital, University of Jena School of Medicine, Jena, Germany

**Keywords:** experimental aesthetics, statistical image properties, self-similarity, fractal dimension, entropy of edge orientations

## Abstract

Several statistical image properties have been associated with large subsets of traditional visual artworks. Here, we investigate some of these properties in three categories of art that differ in artistic claim and prestige: (1) Traditional art of different cultural origin from established museums and art collections (oil paintings and graphic art of Western provenance, Islamic book illustration and Chinese paintings), (2) *Bad Art* from two museums that collect contemporary artworks of lesser importance (© Museum Of Bad Art [MOBA], Somerville, and Official Bad Art Museum of Art [OBAMA], Seattle), and (3) twentieth century abstract art of Western provenance from two prestigious museums (Tate Gallery and Kunstsammlung Nordrhein-Westfalen). We measured the following four statistical image properties: the fractal dimension (a measure relating to subjective complexity); self-similarity (a measure of how much the sections of an image resemble the image as a whole), 1st-order entropy of edge orientations (a measure of how uniformly different orientations are represented in an image); and 2nd-order entropy of edge orientations (a measure of how independent edge orientations are across an image). As shown previously, traditional artworks of different styles share similar values for these measures. The values for *Bad Art* and twentieth century abstract art show a considerable overlap with those of traditional art, but we also identified numerous examples of *Bad Art* and abstract art that deviate from traditional art. By measuring statistical image properties, we quantify such differences in image composition for the first time.

## Introduction

In experimental aesthetics, the search for image features that characterize visual artworks has a long tradition. Already at the inception of this field of research, its founder, Gustav Theodor Fechner (1801–1887), pursued the idea that the golden section plays a role in the aesthetic perception of visual stimuli (Fechner, 1876). Many art critics, philosophers and artists have since postulated that there are universal features in visual artworks that make them beautiful and thus contribute to aesthetic experience (Kandinsky, 1912; Malevich, 1927; Greenberg, 1955; Dowling, 2014). For example, Clive Bell (1881-1964) claimed that artworks possess a “significant form,” which can be universally recognized by humans and persists over time, irrespective of cultural context and depicted content (Bell, 1914).

Diametrically opposed to this view, more recent accounts of aesthetic experience postulate that artworks are defined by contextual factors, such as the historical and social circumstances of their creation, the intentions of the artist and the mode of presentation (Goodman, 1968; Dickie, 1974; Danto, 1981; Bullot and Reber, 2013; Zeki, 2013). In this view, which has dominated most of the last century, the status of an object as an artwork is determined by its ever-changing cultural context and function in society.

The search for image properties that characterize visual artworks has gained new momentum during the last decade, in parallel to technological advances in computer vision and image analysis (Graham and Redies, 2010; Galanter, 2012). Moreover, the focus of vision research has shifted from local image features, such as luminance contrast or edge orientations, to more global image features and their neural underpinnings in the human visual system. Examples are long-distance interactions beyond the receptive field or the sparse (efficient) coding of visual input (Vinje and Gallant, 2002; Simoncelli, 2003). In contrast to local features, global processing can be related in a more straightforward way to the perception of visual beauty in artworks (Redies, 2015; Renoult et al., 2016). We define visual beauty as a sensual property of artworks (not of the objects or persons depicted in the artworks; Redies, 2015). Formalist aesthetic models claim that visual beauty results from a particular spatial arrangement of the pictorial elements in artworks (see above). This arrangement may relate to what vision scientists have called “good Gestalt” or “visual rightness” of artworks (Arnheim, 1954; Locher et al., 1999).

A number of global statistical image properties have been studied in artworks. The following properties, which we focus on in the present work, may serve to illustrate this point. Measures for these properties are described in more detail in the Methods section.

(1) Complexity-related properties. Several statistical image properties relate to the perceived complexity of images, for example, the fractal dimension, the slope of the log-log Fourier spectral plots, the GIF compression rate, edge density, and the strength of luminance gradients. Berlyne found that humans prefer visual patterns with an intermediate degree of complexity (Berlyne, 1974). This finding has been confirmed by other researchers (Forsythe et al., 2011; Taylor et al., 2011), also for paintings of Western provenance (Braun et al., 2013). However, recent studies revealed a considerable inter-individual variability in the preference for complexity (Bies et al., 2016; Güclütürk et al., 2016).

(2) Self-similarity. An image can be considered self-similar if its parts have a structure similar to the image as a whole (Amirshahi et al., 2012). Typically, paintings of Western provenance show an intermediate to high degree of self-similarity. However, self-similarity is not as high in artworks in general as in some natural growth patterns (Braun et al., 2013; Brachmann et al., 2017).

(3) 1st-order entropy of edge orientations. In a large set of artworks from different cultural backgrounds (Western, Islamic and Chinese), we recently found that the orientations of luminance gradients in a painting are relatively evenly spread across the full spectrum of orientations (Redies et al., 2017), confirming results obtained earlier with another method (Koch et al., 2010).

(4) 2nd-order entropy of edge orientations. By pairwise comparison of edge pairs across larger distances in an image, we found that the orientations of distant edges tend to be independent from each other in traditional artworks. Artworks share this property with many natural growth patterns but not with several categories of photographs of man-made scenes and objects (Redies et al., 2017).

Image properties have been investigated in images of artworks that represented different styles of traditional, various artistic techniques and diverse cultural provenance (Taylor, 2002; Graham and Field, 2007, 2008; Redies et al., 2007, 2017; Taylor et al., 2011; Braun et al., 2013; Melmer et al., 2013; Mather, 2014; Brachmann et al., 2017; Hayn-Leichsenring et al., 2017). It remained unclear, however, how universal these properties are across art styles and periods of visual art. We approached the above question by studying two special types of artworks, namely *Bad Art* and abstract art.

*Bad Art* is used as an acronym for a set of artworks from two museums that have specialized in collecting artworks of lesser importance and, presumably, also of different artistic quality (244 artworks from the Museum Of Bad Art © [MOBA] and 44 artworks from the Official Bad Art Museum of Art [OBAMA]). According to its webpages^1^, the MOBA, founded in 1993 in Boston, MA, is “dedicated to the collection, preservation, exhibition and celebration of bad art in all its forms. […] The pieces in the MOBA collection range from the work of talented artists that have gone awry to works of exuberant, although crude, execution by artists barely in control of the brush.” The motto of the museum is: “Art too bad to be ignored.” Images from the MOBA have already been used before in psychological studies (Vartanian et al., 2005; Nordgren and Dijksterhuis, 2008; Bhargave and Montgomery, 2013; Dyck and Johnson, 2017). The OBAMA, founded in 2008 by Marlow Harris and Jo David at Café Racer in Seattle, WA, is less explicit about the type of art they collect^2^, although their artworks seem to be of a type similar to the MOBA artworks.

In the present study, we refer to the images from the two museums as *Bad Art* images to allude to their origin in the two museums. By doing so, we do not intend to insinuate any judgment with regard to the artistic quality of individual artworks in the collections. Nevertheless, on average, we speculate that, if artworks are characterized by specific image properties, then artworks that differ in artistic quality or aesthetic claim differ also in some of the image properties. A major obstacle for comparing artworks of different artistic quality is the lack of an accepted and uniform definition of this term. Indeed, such a definition seems unreachable in practice because aesthetic experience depends not only on formal statistical properties of an artwork, but also on the content displayed in the artworks, their cultural context and the cognitive state of the observer (see above). Despite this obstacle, it seems generally accepted that some artworks are more important or outstanding than others, also with respect to their artistic quality. Although there may be notable exceptions, the general public would probably agree that, on average, artworks on display in prestigious museums tend to have a higher aesthetic value than unknown artworks by laypersons, who lack artistic talent, training and expertise. We speculate that such differences in artistic quality may be associated, in a subset of works of *Bad Art*, with a lower level of visual beauty, which might show up in some of the statistical image properties (Redies, 2015).

Abstract art from the twentieth century was chosen as an example of (post)modern and contemporary art of Western provenance. In this genre, some artists have deliberately departed from traditional art styles by abandoning the concept of visual beauty as a criterion for artistic quality (Dickie, 1974; Danto, 1981). However, not all (post-)modern artists have disposed of visual beauty in their creations (Redies, 2014). We therefore speculated that, on the one hand, there is a large overlap in image properties between abstract art and traditional art styles, but that, on the other hand, a substantial subset of twentieth century abstract art deviates from traditional art genres in the image properties.

In summary, we compare traditional artworks from prestigious museums and art collections (1) to artworks that are considered of a different (or possibly lower) aesthetic quality on average (*Bad Art*), and (2) to twentieth century abstract works of Western provenance, which includes works by artists who did not intend to endow their creations with visual beauty. Previous studies have succeeded in classifying traditional art styles (e.g., Renaissance, Realism or Impressionism) and artists based on image properties (for example, Günsel et al., 2005; Siddiquie et al., 2009; Wallraven et al., 2009; Tan et al., 2016). To our knowledge, this study is first to apply objective statistical image properties to compare artworks of different artistic claim and intentions.

## Methods

### Image datasets

We used four previously published and two novel datasets of images in our analysis. For an overview of the datasets, their origins, and a reference to exemplary images, see Table 1.

**Table 1:** Datasets of images used in the present study.

**No**.	**Dataset**	***n***	**References/source**	**Example images**
1	Western oil paintings (sixteenth to nineteenth century Western art; Jenaesthetics dataset)	1,629	(Amirshahi et al., 2015)	Figures 5A,B,F,J,K
2	Western graphic art	185	(Redies et al., 2007)	Figures 5C,G,L
3	Islamic book illustrations	238	Set no. 16 from Redies et al. (2017)	Figures 5D,H,M
4	Chinese color paintings	215	Set no. 17 from Redies et al. (2017)	Figures 5E,I,N
5	*Bad Art*	288	Museum Of Bad Art © (MOBA), Official Bad Art Museum of Art (OBAMA)	Figure 6
6	Twentieth century abstract art of Western provenance (Tate)	474	Tate Gallery (online collection)	
7	Twentieth century abstract art of Western provenance (NRW)	98	Kunstsammlung Nordrhein-Westfalen (54 images downloaded from the online collection and 44 photographs; Redies and Gross, 2013)	Figure 7
8	Traditional art	740	185 images randomly selected from each of the datasets no. 1–4	

*No., dataset number; n, number of images in the dataset*.

The following four dataset have been published previously: (i) The Jenaesthetics dataset (Amirshahi et al., 2015; Hayn-Leichsenring et al., 2017) contains 1,629 high-quality images of oil paintings of Western provenance that were made available by art museums on the Wikimedia Commons webpages (Google Art Project; set no. 1 in Table 1). The dataset comprises traditional works from art periods that extend from the Renaissance to Expressionism, but no (post-)modern or contemporary art. The paintings depict various subject matters (for example, urban scenes, landscapes, seascapes, architecture, portraits, still lives and nudes). (ii) A similarly diverse dataset of 185 graphic artworks (monochrome works on paper; set no. 2). This dataset corresponds to a previously published dataset of 200 graphic artworks of Western provenance (Redies et al., 2007), but without the 15 examples of (post-)modern and contemporary art that were contained in the original dataset. To extend our study to traditional artworks from other cultural backgrounds, a dataset of (iii) 238 images of Islamic book illustrations (set no. 3) and (iv) a dataset of 215 images of traditional Chinese color paintings (set no. 4), both from different centuries, were downloaded from the Wikimedia Commons webpages and included in the analysis (Redies et al., 2017). Note that the four datasets of traditional artworks represent a large variety of artistic techniques (oil paintings, prints, drawings, watercolors etc.).

The *Bad Art* dataset (set no. 5) contained 244 digitized reproductions from the MOBA collection, kindly provided by its curator, Mr. Michael Frank. In addition, we downloaded 44 images of artworks from the official web collection of the OBAMA^3^. Only high-resolution images that did not show obvious artifacts (blurring, JPEG artifacts, reflections, etc.) were included in the analysis. If present, frames around the images were removed.

From the website of the Tate Gallery^4^, 474 images of twentieth century abstract art of Western provenance were downloaded between October 22 and November 13, 2016 (set no. 6). Images of similar abstract art were also obtained from the Kunstsammlung Nordrhein-Westfalen (set no. 7). Fifty-four images were downloaded from the collection's website^5^ between November 6 and December 21, 2016, and another 44 images were photographed by one of the authors previously (Redies and Gross, 2013). We used only high-resolution images and restricted the number of artworks by individual artists to avoid overrepresentation of particular types of abstract art. A preliminary analysis showed that the datasets of abstract art images from the two museums did not differ in the statistical values calculated in the present study. We therefore merged the images of abstract art into a single dataset of 572 images for further analysis.

### Image analysis

We determined four statistical image properties by following previously published procedures (Amirshahi et al., 2012; Braun et al., 2013; Redies et al., 2017). Briefly, the properties were calculated as follows:

#### Fractal dimension

In the present work, the fractal dimension was determined with the box-counting method (Taylor et al., 1999) after applying a canny-edge filter to each image to obtain binary (edge) images. In 2d space, curves or patterns have a fractal dimension between 1 (low complexity) and 2 (high complexity). For a detailed description of the method, see Redies et al. (2015).

#### Self-similarity of gradient orientations

Self-similarity was calculated with a method that was derived from the Pyramid Histogram of Oriented Gradients (PHOG) (Bosch et al., 2007), as described before (Amirshahi et al., 2012). In brief, each color image was transformed into the Lab color space and reduced by isotropic scaling and bicubic interpolation to a size of 100,000 pixels. We then generated histograms of oriented luminance gradients (HOG features, Dalal and Triggs, 2005) for each image at consecutive levels of an image pyramid up to level 3 of the pyramid. A measure of self-similarity was derived from a comparison of the histograms at different levels of the pyramid with the ground level histogram. Self-similarity is higher (closer to 1) if the histograms at different levels of the pyramid are more similar to the histogram at the ground level. A value of 0 indicates minimal self-similarity. A detailed description of the method can be found in the Appendix to Braun et al. (2013).

#### First-order and 2nd-order entropy of edge orientations

The spatial distribution of the edge orientations across each image was studied by determining the Shannon entropy of 1st-order and 2nd-order edge orientation histograms (for a detailed description, see Redies et al., 2017). After scaling down the high-resolution input images to a maximum size of 120,000 pixels, color images were converted to grayscale using an algorithm that weights color channels according to their perceived luminosity (ITU-R-601-2 luma transform). Then, edges were extracted by applying a bank of 24 oriented Gabor filters, which covered one full rotation when combined. Because computational limitations ruled out the comparison of all edges with all other edges in an image, only the 10,000 highest edge responses were analyzed for each image.

As a measure of how uniformly the edge orientations were distributed across the full spectrum of orientations, we calculated the Shannon entropy of the histograms that summed up the strength of all edge orientations across the entire image (*1st-order entropy* of edge orientations).

As a measure of how independent edge orientations are across an image, we calculated the *2nd-order entropy* of edge orientations. The orientation of each edge was related pairwise to the orientation of all other edges (Geisler et al., 2001; Redies et al., 2017). Close-by edge pairs (distance less than 20 pixels) were excluded from the analysis to avoid local regularities, such as collinearity (Geisler et al., 2001; Sigman et al., 2001; Redies et al., 2015). Histograms of the orientation differences were then obtained for all distances between the edge pairs in an image. Second-order Shannon entropy in these histograms is maximal if all orientation differences occurred at equal strength in the histograms. In this case, the orientation of a given edge does not allow predicting the orientation of other edges in the image, i.e., edge orientations are independent of each other across an image.

### Statistical analysis

Non-parametric tests were used throughout the analysis because the values for most measures were not normally distributed. Means for different image categories were compared by a one-way ANOVA (Kruskal-Wallis test, followed by Dunn's post-test). A level of *p* < 0.05 was considered significant. Effect sizes (η^2^) of differences between pairs of image categories were calculated by the Wilcoxon rank-sum test. In the box plots, the whiskers bracket 5–95% of the data.

## Results

We measured four global statistical image properties (see Introduction) in artworks and compared traditional art of high artistic claim to artworks of lesser importance (*Bad Art*), and to abstract art (Table 1).

### Differences in image properties

**Figure 1:**
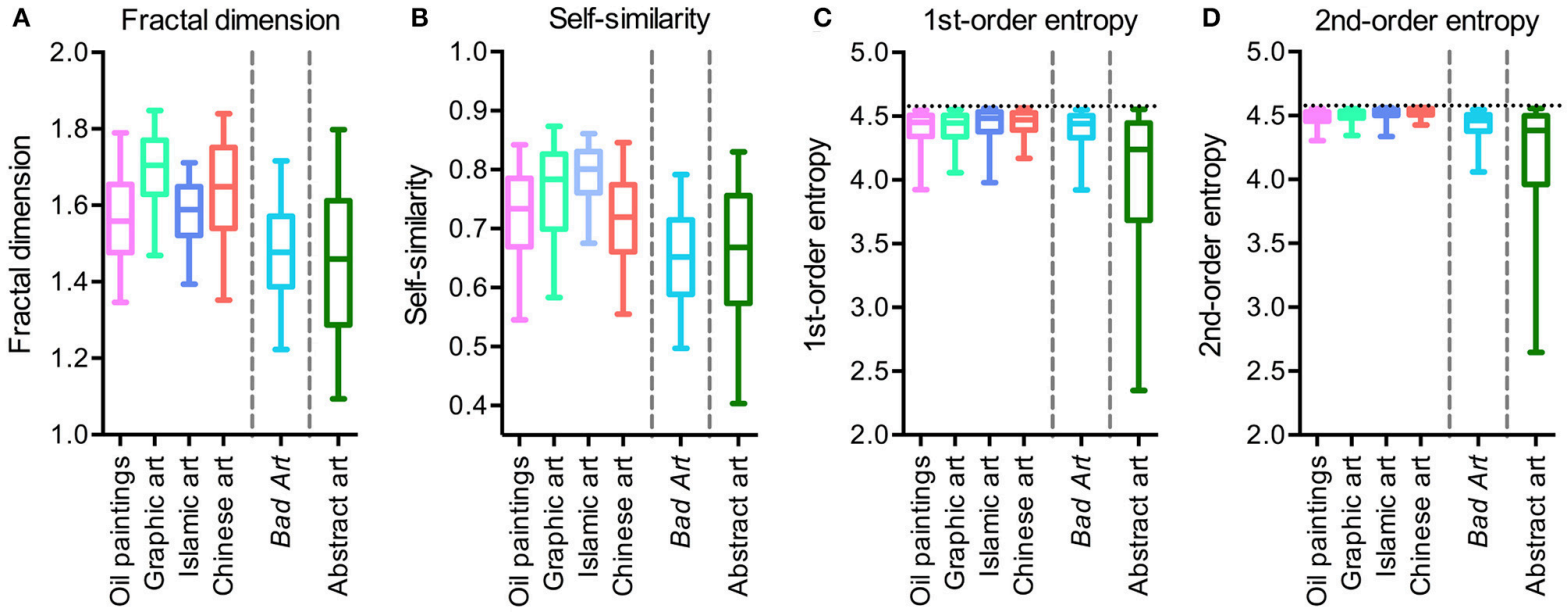
Box plots of the fractal dimension **(A)**, self-similarity **(B)**, 1st-order edge entropy **(C)** and 2nd-order edge entropy **(D)** for the different image categories analyzed. The effect sizes and significance levels of the differences between the median values are listed in Tables 2, 3.

**Figure 2:**
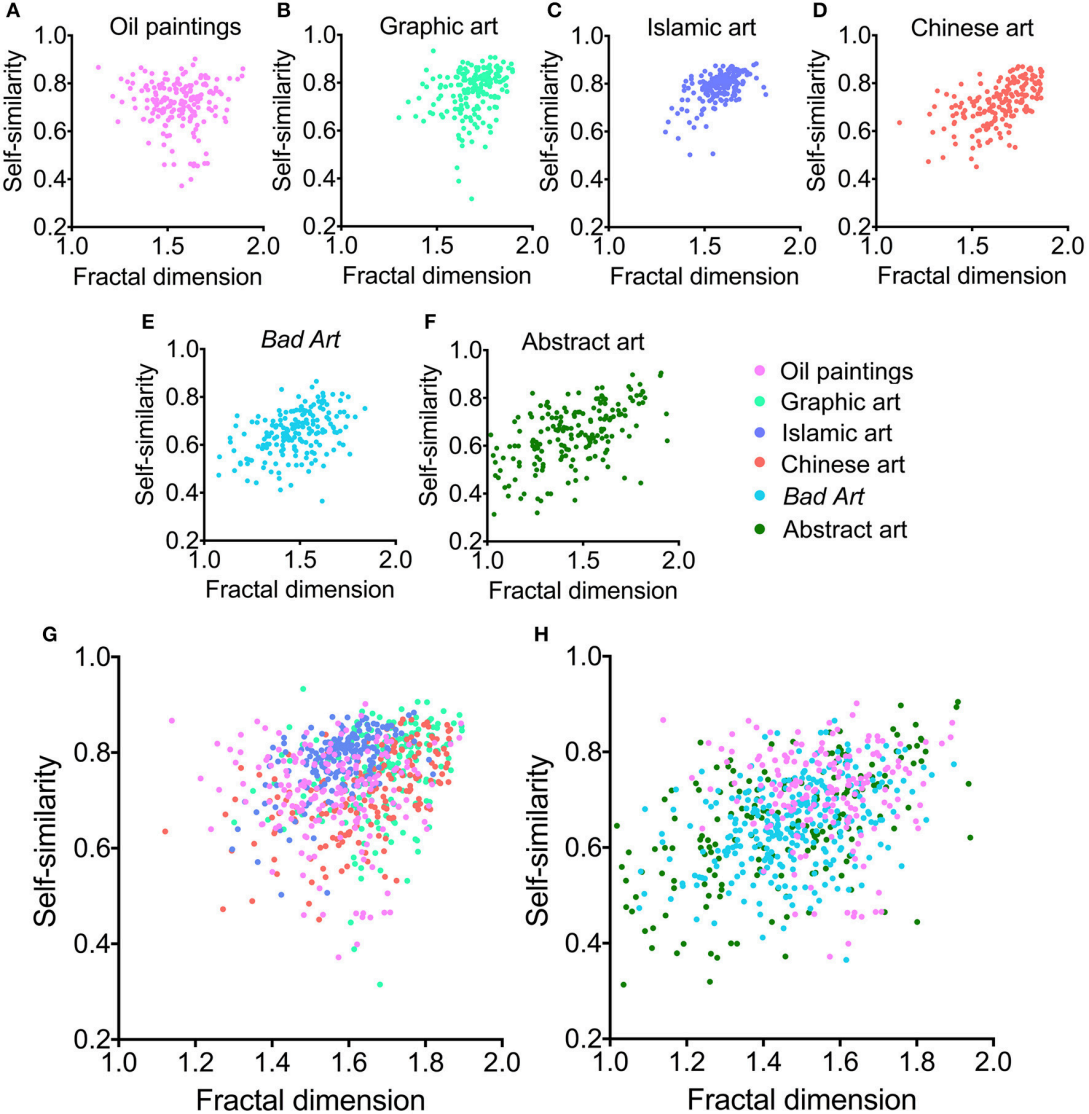
**(A–F)** Scatter plots of fractal dimension and self-similarity for the different image categories analyzed. Each dot represents one of the 185 images that were randomly selected from each category. **(G)** Overlay of results for traditional art (Western oil paintings, Western graphic art, Islamic art, and Chinese art) and **(H)** for Western oil paintings, *Bad Art* and abstract art. The color coding is indicated to the right-hand side of **(F)**.

**Figure 3:**
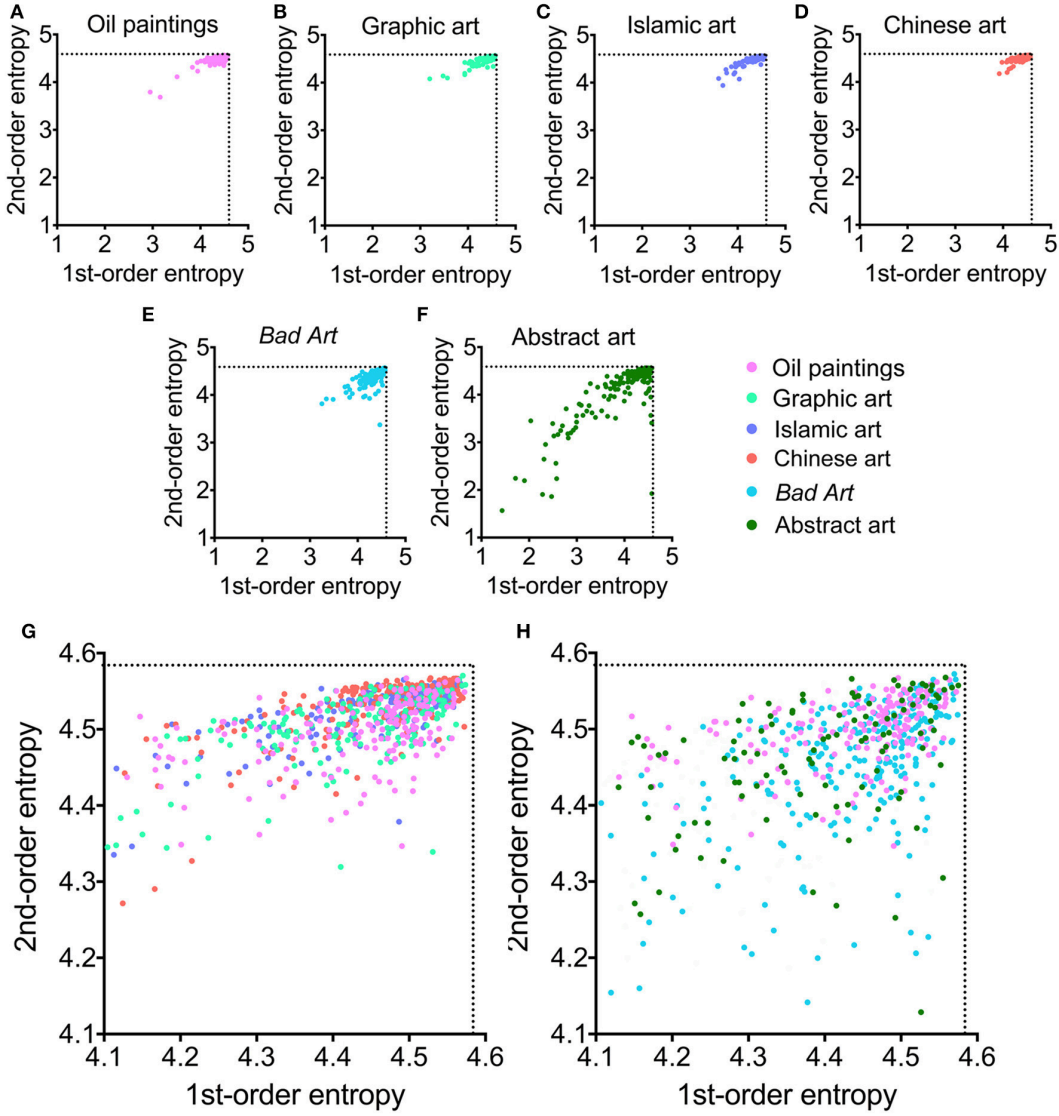
**(A–F)** Scatter plots of 1st-order entropy and 2nd-order entropy of edge orientations for the different image categories analyzed. Each dot represents one of the 185 images that were randomly selected from each category. **(G)** Overlay of results for traditional art (Western oil paintings, Western graphic art, Islamic art, and Chinese art). **(H)** Overlay of results for Western oil paintings, *Bad Art* and abstract art. Note that **(G,H)** are higher magnifications of the upper right corners of the plots shown in **(A–F)**. The color-coding is indicated to the right-hand side of **(F)**.

Figure 1 shows box plots for the four image properties for all datasets of artworks. Mean values and standard deviations are listed in Table 2. We observed significant differences between the six image categories (Kruskal-Wallis test) for the fractal dimension (*H* = 423, *df* = 5, *p* < 0.0001), self-similarity (*H* = 471, *df* = 5, *p* < 0.0001), 1st-order entropy (*H* = 278, *df* = 5, *p* < 0.0001) and 2nd-order entropy of edge orientations (*H* = 544, *df* = 5, *p* < 0.0001). Effect sizes (η^2^; Wilcoxon rank-sum test) for the differences between the individual image categories for the fractal dimension and self-similarity are listed in Table 3, and for 1st-order and 2nd-order edge entropy in Table 4. In Figures 2, 3, the values for 185 randomly selected images from each dataset are plotted in 2d scatter diagrams.

**Table 2:** *Mean* values ± *S.D*. for the statistical properties of all datasets of art images. *n*, number of images.

**Image dataset**	**No. in Table 1**	**Fractal dimension**	**Self-similarity**	**1st-order entropy**	**2nd-order entropy**
Western oil paintings (*n* = 1,629)	1	1.56 ± 0.13	0.72 ± 0.09	4.380 ± 0.214	4.474 ± 0.100
Western graphic art (*n* = 185)	2	1.69 ± 0.11	0.76 ± 0.10	4.391 ± 0.189	4.489 ± 0.079
Islamic book illustrations (*n* = 238)	3	1.58 ± 0.10	0.79 ± 0.06	4.416 ± 0.180	4.506 ± 0.085
Chinese color paintings (*n* = 215)	4	1.63 ± 0.15	0.71 ± 0.09	4.437 ± 0.122	4.519 ± 0.055
*Bad Art* (*n* = 288)	5	1.47 ± 0.15	0.65 ± 0.13	4.371 ± 0.234	4.408 ± 0.177
Twentieth century abstract art (*n* = 572)	6 + 7	1.45 ± 0.22	0.65 ± 0.13	3.945 ± 0.722	4.093 ± 0.672

**Table 3:** Effect size (η^2^) and significance levels (Wilcoxon rank-sum test) for differences between the categories of artworks in the fractal dimension (upper right side of table) and self-similarity (lower left side).

**Category of artworks**	**Western oil paintings**	**Western graphic art**	**Islamic book illustrations**	**Chinese color paintings**	***Bad Art***	**Twentieth century abstract art**
Western oil paintings	_	0.084^****^	n.s.	0.028^****^	0.043^****^	0.055^****^
Western graphic art	0.023^****^	_	0.254^****^	0.037^***^	0.404^****^	0.244^****^
Islamic book illustrations	0.081^****^	0.014^*^	_	0.058^****^	0.148^****^	0.086^****^
Chinese color paintings	n.s.	0.082^****^	0.210^****^	_	0.223^****^	0.146^****^
*Bad Art*	0.078^****^	0.292^****^	0.494^****^	0.118^****^	_	n.s.
Twentieth century abstract art	0.052^****^	0.142^****^	0.273^****^	0.040^****^	n.s.	_

Significance levels

* p < 0.05,

*** p < 0.001,

**** *p < 0.0001 (Kruskal-Wallis test with Dunn's post-test)*.

**Table 4:** Effect size (η^2^) and significance levels (Wilcoxon rank-sum test) for differences between the categories of artworks in 1st-order entropy (upper right side of table) and 2nd-order entropy (lower left side).

**Category of artworks**	**Western oil paintings**	**Western graphic art**	**Islamic book illustrations**	**Chinese color paintings**	***Bad Art***	**Twentieth century abstract art**
Western oil paintings	_	n.s.	0.009^****^	0.007^***^	n.s.	0.096^****^
Western graphic art	0.004^**^	_	0.018^**^	0.016^*^	n.s.	0.097^****^
Islamic book illustrations	0.048^****^	0.073^****^	_	n.s.	0.024^***^	0.153^****^
Chinese color paintings	0.059^****^	0.099^****^	n.s.	_	0.022^***^	0.157^****^
*Bad Art*	0.034^****^	0.116^****^	0.250^****^	0.300^****^	_	0.104^****^
Twentieth century abstract art	0.118^****^	0.147^****^	0.252^****^	0.274^****^	0.044^**^	_

Significance levels

* p < 0.05,

** p < 0.01,

*** p < 0.001,

**** *p < 0.0001 (Kruskal-Wallis test with Dunn's post-test)*.

The median values of the fractal dimension (Figure 1A and Table 3) for *Bad Art* and abstract art are lower than for all of the four traditional art styles. Differences are observed also between the traditional art styles (Kruskal-Wallis test; *H* = 195, *df* = 3, *p* < 0.0001). The highest median value is observed for graphic art. *Bad Art* does not differ from abstract art in this measure.

Self-similarity (Figure 1B and Table 3) is also lower for *Bad Art* and abstract art than for the traditional art categories. The traditional art styles differ from each other (*H* = 186, *df* = 3, *p* < 0.0001), except for Western oil paintings and Chinese paintings. Self-similarity values are highest for the graphic artworks and the Islamic artworks.

For 1st-order and 2nd-order entropy of edge orientations (Figures 1C,D and Table 4), values of abstract art are widely scattered, ranging from very low to near maximal values. Mean values of 1st-order edge entropy for *Bad Art* are slightly lower than those for the Islamic and Chinese art but similar to the mean values for the oil paintings and graphic artworks. Mean values of 2nd-order edge entropy are higher for *Bad Art* than for abstract art but lower than the mean values for all categories of traditional art. Some of the traditional art categories also differ from each other (1st-order entropy, *H* = 28, *df* = 3, *p* < 0.0001; 2nd-order entropy, *H* = 182, *df* = 3, *p* < 0.0001).

The scatter diagrams visualize these findings in more detail. For traditional artworks, data for the fractal dimension and self-similarity are concentrated toward the upper right corner of the plots (Figures 2A–D); values are high for both measures and the data points overlap extensively (Figure 2G). *Bad Art* and abstract art (Figures 2E,F,H) also overlap with traditional art, but a larger proportion of data points scatters toward the lower left corner of the plots, i.e., they show lower values for both measures.

Similar observations can be made for the scatter plots of 1st-order and 2nd-order edge entropy (Figure 3). Close to maximal entropy values are found for all four categories of traditional art (Figures 3A–D,G). For 1st-order edge entropy, the data points of *Bad Art* (Figures 3E,H) overlap with the traditional art categories, especially with Western oil paintings and graphic art. For 2nd-order edge entropy, there is also a considerable overlap, but a larger proportion of *Bad Art* data points are low compared to the other art styles (Figures 3E,H). For abstract art (Figures 3F,H), we again observe some overlap, but even more data points are found at lower values for both 1st-order entropy and 2nd-order entropy (Figures 3F,H).

In conclusion, there is a considerable overlap between all six art categories. However, *Bad Art* and, even more so, abstract art differ in the measured average image properties from most of the traditional art styles. Moreover, the dataset of abstract artworks contains images that scatter widely in their values for 1st-order entropy and 2nd-order entropy of edge orientations (Figures 1C,D; see also *S.D*. values in Table 2).

### Mahalanobis distance

To quantify the overall differences between the artworks of the different categories, we calculated their Mahalanobis distances to reference datasets in the 4d space that is spanned by the image properties studied in the present experiment. Specifically, we calculate the distances of each image in a given category to the median of one of the other categories (here called the *reference dataset*) by a pairwise comparison between image categories. Note that we can calculate the Mahalanobis distance, as a baseline, also between images of one category and the median of that same category. Because the values for none of the image categories passed the D'Agostino and Person omnibus normality test, we calculated median values and median absolute deviations (*M.A.D.s*) for the Mahalanobis distances (Table 5).

**Table 5:** Median Mahalanobis distances between the categories of art images (± median absolute deviation).

	**Western oil paintings**	**Western graphic art**	**Islamic book illustrations**	**Chinese color paintings**	***Bad Art***	**Twentieth century abstract art**
Western oil paintings	1.55 ± 0.72	2.02 ± 0.97^****^	2.47 ± 1.29^****^	2.00 ± 1.01^****^	1.69 ± 0.77^*^	1.19 ± 0.41^****^
Western graphic art	1.84 ± 0.61^****^	1.52 ± 0.71	2.54 ± 1.04^****^	1.97 ± 0.65^****^	2.25 ± 0.70^****^	1.54 ± 0.34
Islamic book illustrations	1.46 ± 0.47	1.65 ± 0.60	1.37 ± 0.64	1.87 ± 0.62^**^	2.02 ± 0.49^****^	1.32 ± 0.30^***^
Chinese color paintings	1.65 ± 0.65	1.65 ± 0.62	2.73 ± 1.23^****^	1.59 ± 0.55	1.82 ± 0.79^*^	1.33 ± 0.43^**^
*Bad Art*	1.98 ± 1.01^****^	2.81 ± 1.36^****^	3.59 ± 1.70^****^	2.53 ± 1.32^****^	1.54 ± 0.70	1.11 ± 0.36^****^
Twentieth century abstract art	2.88 ± 2.25^****^	3.80 ± 3.32^****^	4.43 ± 3.13^****^	4.16 ± 3.76^****^	2.55 ± 1.64^****^	1.44 ± 0.68

The artwork categories listed on the top of the columns represent the reference datasets for the calculation of the distances. Significance levels

* p < 0.05;

** p < 0.01;

*** p < 0.001;

**** *p < 0.0001 (Kruskal-Wallis test with Dunn's post-test)*.

**Figure 4:**
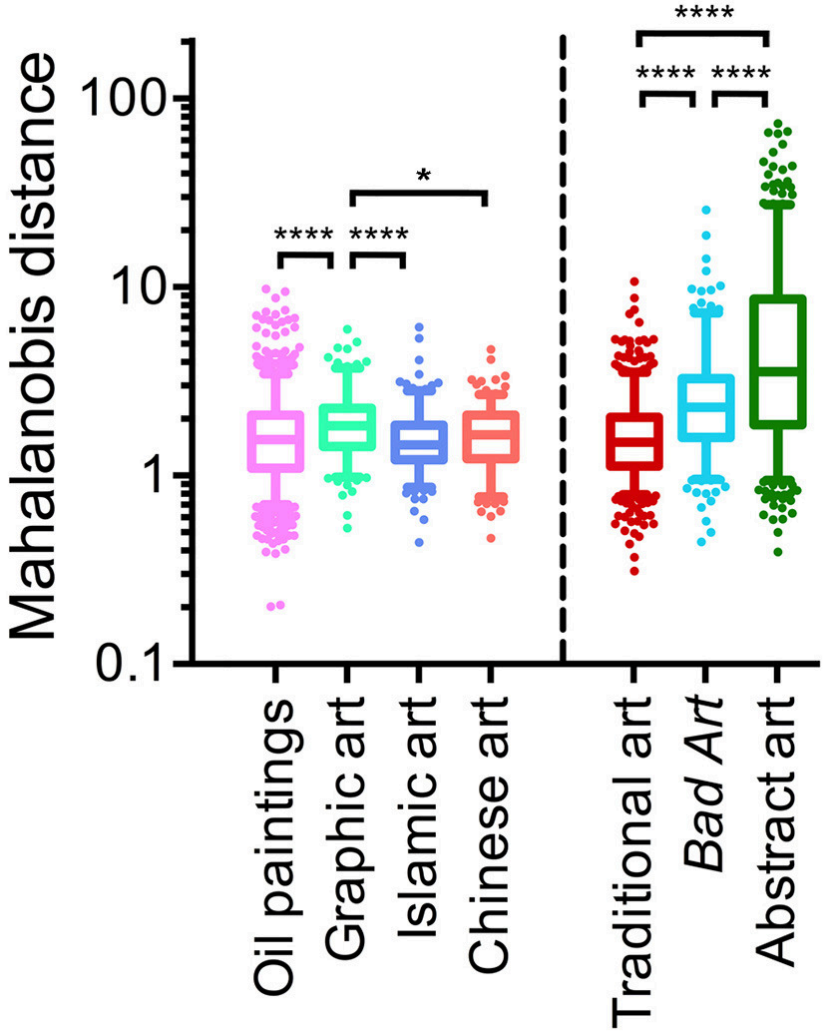
Box plots of the Mahalanobis distances. The *left-hand side* shows distances of the individual traditional artworks (1629 images of Western oil paintings, 185 images of Western graphic art, 238 images of Islamic art, 215 images of Chinese art) to the median value of all oil paintings. The *right-hand side* shows distances of the traditional artworks (*red*; 185 randomly selected images each of oil paintings, graphic art, Islamic art, and Chinese art), *Bad Art* (288 images) and abstract artworks (572 images) to the median value of the traditional artworks. Significance levels *p < 0.05, ****p < 0.0001 (Kruskal-Wallis test with Dunn's post-test).

Figure 4 shows box plots of the results. For the left-hand part of the figure, the reference dataset are the Western oil paintings. Distances of all four traditional art datasets to the Western oil paintings are shown. Western graphic art has distances larger than the median distance of oil paintings to themselves, whereas the distances of Islamic and Chinese art to the Western oil paintings are of similar magnitude. For the right-hand part of the Figure 4, a dataset of 185 images that were randomly selected from each of the four traditional art categories (740 images in total; dataset no. 8 in Table 1) served as the reference dataset (*red* box plot in Figure 4). On average, distances are larger for *Bad Art* and, in particular, for the abstract artworks than the distances of images in the traditional artwork dataset to itself. Mean distances for all possible pairwise comparisons between the six original datasets are summarized in Table 5.

As a measure of the dispersion of the distances, we calculated the median absolute deviation of each distance to the median distance of the reference dataset for each comparison. Results in Table 5 confirm that the deviations are larger for the comparisons of *Bad Art* and abstract art to the traditional artwork categories, respectively, than for the comparisons between the traditional artworks (Figure 4).

In conclusion, as described previously (Redies et al., 2017), we find that, to some extent, artworks of Western, Islamic and Chinese origin share a specific pattern of statistical image properties. The image properties of *Bad Art* and abstract art considerably overlap with this pattern. At the same time, a large number of *Bad Art* and abstract artworks deviate considerably from the traditional art categories; these deviations are more pronounced for abstract art than for *Bad Art*.

### Example images

**Figure 5:**
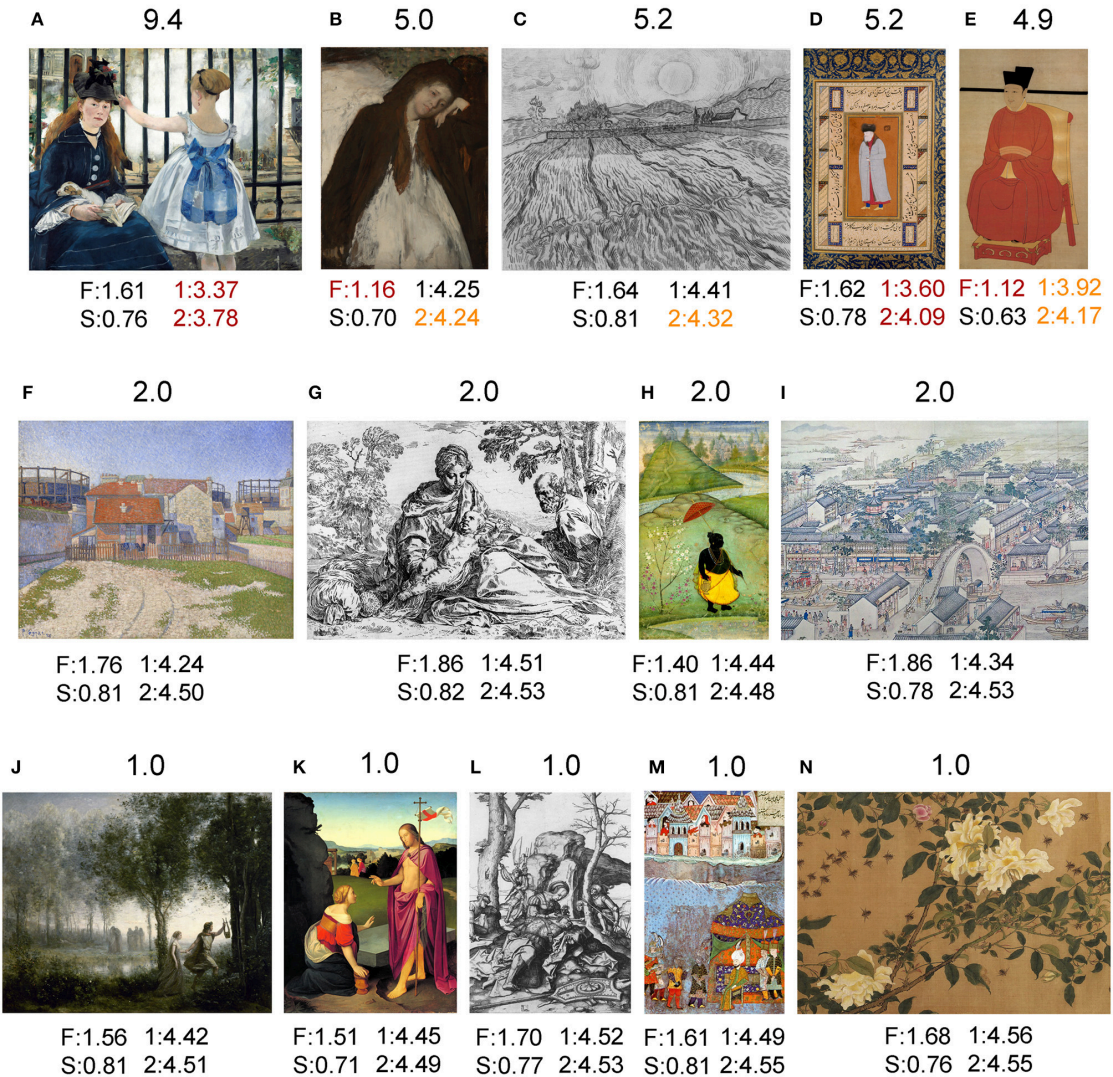
Results for examples of traditional art images **(A,B,F,J,K**, Western oil paintings; **C,G,L**, Western graphic art; **D,H,M**, Islamic book illustrations; **E,I,N**, Chinese color paintings). Above each image, its Mahalanobis distance to the median value of traditional artworks (*red* in Figure 4; 185 randomly selected images each of oil paintings, graphic art, Islamic art, and Chinese art, respectively) is shown. Below each image, its values for the fractal dimension (F), self-similarity (S), 1st-order entropy (1) and 2nd-order entropy (2) are displayed. *Orange* indicates values that lie outside the 5–95% data interval and *red* indicates values that lie outside the 1–99% data interval. The following artworks are shown: **(A)** Édouard Manet, Le Chemin de Fer, 1873; **(B)** Edgar Degas, The Convalescent, 1872–1887; **(C)** Vincent van Gogh, Wheat Field With Rising Sun, 1889; **(D)** Muhammadi, Portrait of Russian Ambassador, 1580s; **(E)** portrait painting by Zhao Ji (1082–1135); **(F)** Paul Signac, Les Gazomètres. Clichy, 1886; **(G)** Somine Cantarini, Rest on the Flight to Egypt, 1640; **(H)** Unknown artist (Mughal Style), Vamana, 1610; **(I)** Xu Yang, Prosperous Suzhou, 1759 (detail of scroll), **(J)** Camille Corot, Orphée Ramenant Eurydice des Enfers, 1861; **(K)** Friedrich Overbeck, Easter Monday, 1818; **(L)** Lucas van Leyden, Samson and Delilah, 1508; **(M)** Unknown Turkish artist, Surrender of Becskerek, sixteenth century; and **(N)** Zhao Chang (959–1016), Yellow Roses and Bees, Pink Roses and Wasps (detail of scroll). All images shown in this figure are in the public domain.

**Figure 6:**
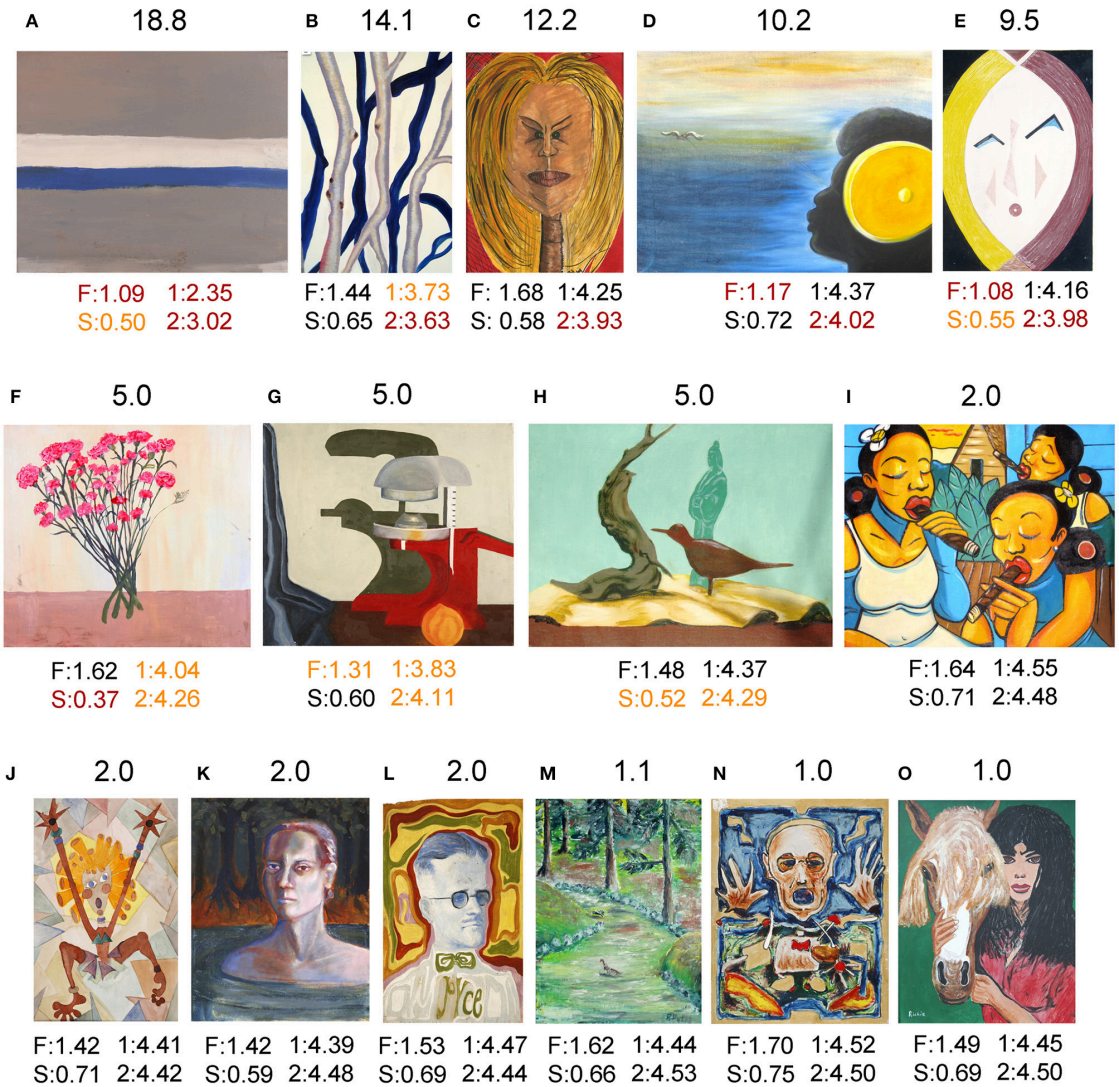
Results for examples of *Bad Art* images from the © Museum Of Bad Art, Somerville, MA. Above each image, the Mahalanobis distance to the median value of traditional artworks (*red* in Figure 4; 185 randomly selected images each of oil paintings, graphic art, Islamic art, and Chinese art, respectively) is shown. Below each image, values for the fractal dimension (F), self-similarity (S), 1st-order entropy (1) and 2nd-order entropy (2) are displayed. *Orange* indicates values that lie outside the 5–95% data interval and *red* indicates values that lie outside the 1–99% data interval. The following artworks are shown: **(A)** Anonymous, Study in Blue Gray and White; **(B)** Anonymous, Birch Shadow; **(C)** Anonymous, Hollywood Lips; **(D)** Anonymous, Flap; **(E)** Anonymous, You Like Me I Like You; **(F)** Elisabeth Angelozzi, No Visible Means of Support; **(G)** Anonymous, Still Life With Juicer; **(H)** Anonymous, Still Life With Jade; **(I)** Anonymous, Mujeres Y Puros Hermosos; **(J)** M. Starbuck, Yikes!; **(K)** Anonymous, Ashen Woman Rising; **(L)** Anonymous, Portrait of the Artist as a Blue Man; **(M)** Anonymous, Chicken Crossing the Road; **(N)** Mark Gewiss, To Life, 1993; and **(O)** Anonymous, Think Again. The images are reproduced with kind permission from © Museum Of Bad Art, Somerville, MA.

**Figure 7:**
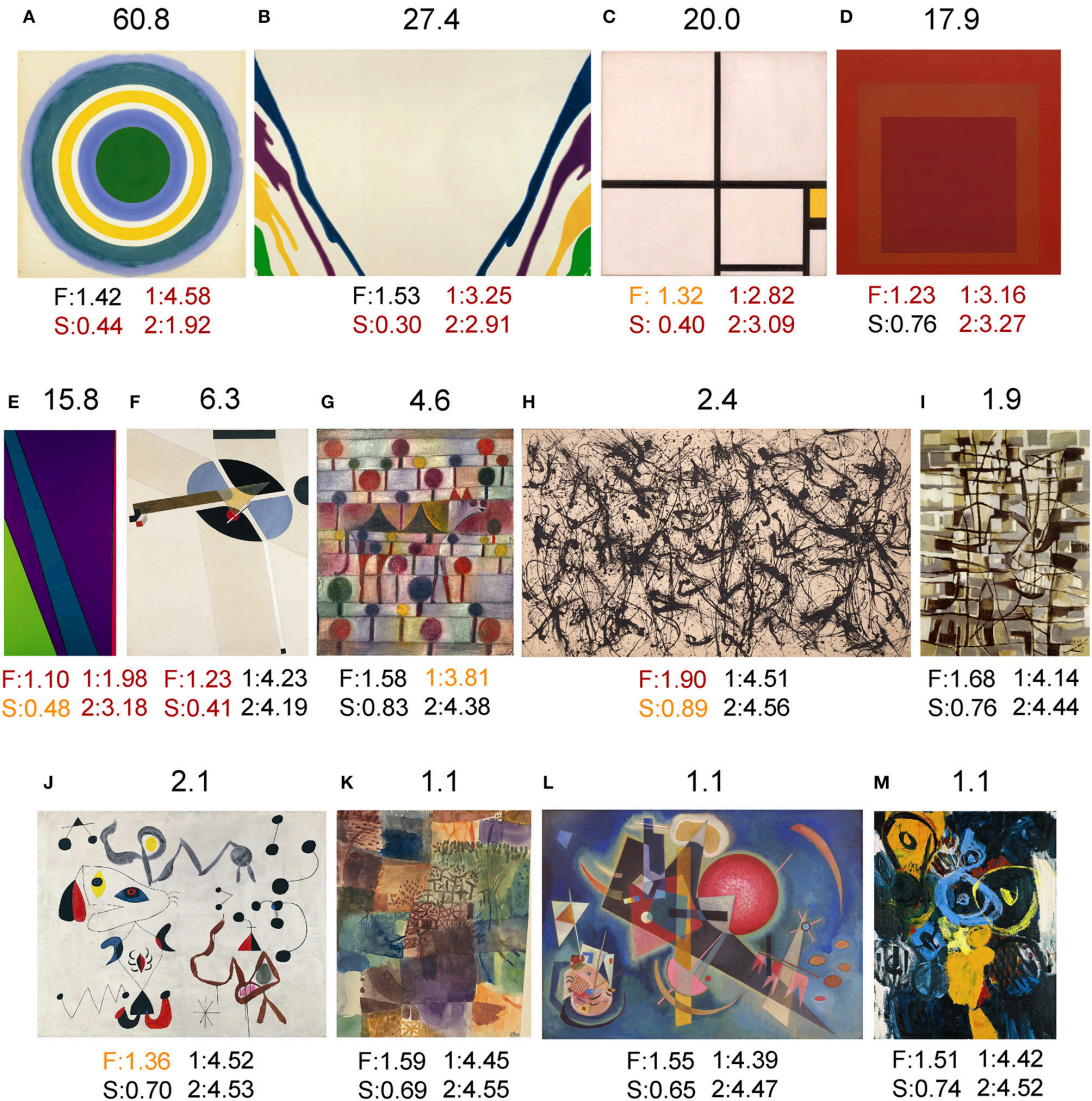
Results for examples of twentieth century *abstract art* of Western provenance from the Kunstsammlung Nordrhein-Westfalen. Above each image, the Mahalanobis distance to the median value of traditional artworks (*red* in Figure 4; 185 randomly selected images each of oil paintings, graphic art, Islamic art and Chinese art, respectively) is shown. Below each image, the values for the fractal dimension (F), self-similarity (S), 1st-order entropy (1) and 2nd-order entropy (2) are displayed. *Orange* indicates values that lie outside the 5–95% data interval and *red* indicates values that lie outside the 1–99% data interval. The following artworks are shown: **(A)** Kenneth Noland, Bloom, 1960; **(B)** Morris Louis, Gamma Gamma, 1959-1960; **(C)** Piet Mondrian, Composition in Yellow, 1930; **(D)** Josef Albers, Homage to the Square: Red Va, 1967; **(E)** Olle Baertling, Ayaru, 1972; **(F)** El Lissitzky, Proun G 7, 1923; **(G)** Paul Klee, Kamel (in rhythm). Baumlandschaft, 1920/1943; **(H)** Jackson Pollock, Number 32, 1950; **(I)** Jean Bazaine, Lune et Oiseau de nuit, 1947; **(J)** Joan Miró, Femmes et Oiseaux dans la Nuit, 1945; **(K)** Paul Klee, Erinnerung an einen Garten, 1914/1917; **(L)** Wassily Kandinsky, In the Blue, 1925; and **(M)** Ernst Wilhelm Nay, Schlüsselzeichen, 1962. The images shown in **(A,B,D,E,H,I,J,M)** are reproduced with kind permission from © VG Bild-Kunst, Bonn 2017. These and the other images **(C,F,G,K,L)** were kindly reproduced with permission from the Kunstsammlung Nordrhein-Westfalen.

To get a better visual grasp of some of the differences described above, example images are shown for traditional art in Figure 5, for *Bad Art* in Figure 6, and for abstract art in Figure 7. Proceeding from the upper left part to the lower right part of the figures, images are shown for Mahalanobis distances to the center of traditional artworks (*red* in Figure 4) above 5, then around 5, to around 2 and 1, which is close to the median distance of traditional artwork to themselves.

Of the most deviant traditional artworks, the Manet painting (Figure 5A), shows a prominent railing in the background with bars that are oriented in parallel and result in low entropy values. The Degas painting (Figure 5B) and the Chinese portrait painting (Figure 5E) display large homogeneous areas that are delineated by long and relatively straight edges, resulting in a low fractal dimension and low 2nd-order edge entropy, respectively. In the van Gogh drawing (Figure 5C), lines are predominantly oriented in parallel (low 2nd-order edge entropy). In the Islamic book illustration (Figure 5D), cardinal orientations prevail (low entropy values). These characteristics set the images apart from the traditional artworks with more typical values (Figures 5F–N).

Images of *Bad Art* (Figure 6) can differ from traditional artworks with respect to several statistical properties. For example, a low fractal dimension, which corresponds to the impression of low image complexity, is observed in the images shown in Figures 6A,D,E,G. In some other images, the prominence of particular orientations manifests itself in low values for edge entropy (Figures 6A,B,F,G). Examples of artworks with values similar to traditional artworks are shown in Figures 6I–O.

For some examples of twentieth century abstract art of Western provenance (Figure 7), deviating values can be directly related to their style and visual appearance. For example, the painting by Kenneth Noland (Figure 7A) shows close to maximal 1st-order entropy of edge orientations because all orientations are present at equal strength due to the strictly circular structure of the painting. At the same time, 2nd-order edge entropy is very low because of its high co-circularity (Sigman et al., 2001). In the paintings depicted in Figures 7B–E, both 1st-order entropy and 2nd-order entropy are low because the range of orientations is rather restricted. This deviation from traditional artworks is particularly evident in the works by Piet Mondrian (Figure 7C) and Josef Albers (Figure 7D), which consists of horizontal and vertical orientations only. Self-similarity is low in paintings that exhibit large homogeneous areas (Figures 7A–C,E,F). There are also many abstract artworks with values well within the traditional art category (Figures 7I–M), including works by Joan Miró (Figure 7J), Paul Klee (Figure 7K), and Wassily Kandinsky (Figure 7L). Interestingly, the drip paintings by Jackson Pollock (Figure 7H) resemble traditional paintings, except for a fractal dimension and self-similarity that are higher than for most other abstract and traditional artworks.

## Discussion

By analyzing four statistical image properties (fractal dimension, self-similarity, and 1st order entropy and 2nd-order entropy of edge orientations), we obtained similar values for sets of traditional artworks from different cultural backgrounds (Western, Islamic and Chinese). Corresponding values for the datasets of *Bad Art* and abstract art images scattered more widely, but overlapped considerably with the values of the traditional artworks. However, we also found many examples of *Bad Art* and abstract art that deviated from the values of traditional art.

### Traditional artworks share image properties across cultures

The present work confirms previous results that large sets of traditional artworks share specific statistical image properties on average. Examples for common properties are the Fourier spectral slope (Graham and Field, 2007, 2008; Redies et al., 2007; Melmer et al., 2013; Mather, 2014), self-similarity (Hayn-Leichsenring et al., 2017), edge orientation entropy (Redies et al., 2017) and variance of filter responses (Brachmann et al., 2017). Similarities have been found both across cultures (Western, Islamic and Chinese) (Graham and Field, 2008; Melmer et al., 2013; Redies et al., 2017), for different genres of Western Art and for different content depicted in artworks (Redies et al., 2007, 2017; Hayn-Leichsenring et al., 2017). A comparison of traditional artworks with various non-art images (natural or artificial objects, patterns and scenes) confirmed that the image properties are characteristic for traditional artworks. These findings are in line with the general suggestion that visual artworks represent stimuli with specific perceptual properties that are neither arbitrary nor random (Fechner, 1876; Bell, 1914; Dowling, 2014; Redies, 2015; Renoult et al., 2016). These properties might contribute to the “significant form” that has been postulated by Bell (1914). However, there are varying degrees of overlap with the properties of natural patterns and scenes (see Introduction). Such similarities provided the basis of ideas on how the perception of artworks is linked to the processing of natural scenes and objects, to which the visual system is adapted in evolution and development (Orians, 1986; Hodgson, 2006; Redies, 2007; Graham and Meng, 2011; Taylor et al., 2011). These ideas should be viewed as complementary, not as contradictory (Hodgson and Verpooten, 2015; Redies, 2015) to concepts that view visual art as a cultural phenomenon wherein contextual and conceptual factors play a central role (Dickie, 1974; Danto, 1981). Despite these overall similarities across cultures and art genres, there is a considerable inter-individual variability in the preference for statistical image properties within groups of people of similar cultural background, both for art and non-art stimuli (for example, see Jacobsen, 2004; Mallon et al., 2014; Bies et al., 2016; Güclütürk et al., 2016; Lyssenko et al., 2016).

### Comparison of traditional art to *Bad Art* and abstract art

The present results confirm our initial expectation that (1) there is a large overlap between all six art genres investigated in the present study, and (2) many images of *Bad Art* and (post-)modern abstract art of Western provenance differ in their statistical properties from traditional art from prestigious museums. As a quantitative corroboration of these findings, we calculated the Mahalanobis distances between the artworks in the multidimensional space that is spanned by the four image properties. In this space, the distances from the *Bad Art* and abstract art images to the traditional artworks are larger on average than the distances of the traditional artworks to themselves (Figure 4). Moreover, the scatter of the values for *Bad Art* and abstract art is large compared to that for traditional art (Table 5).

In the case of *Bad Art*, the deviating examples (Figures 6A–H) show several stylistic peculiarities not found in most traditional artworks, such as a predominance of particular orientations (resulting in low edge orientation entropy), large homogeneous areas (low self-similarity) and low complexity (low fractal dimension).

For some images of twentieth century abstract art, similar deviations were observed (Figures 7A–G). For example, the strictly circular painting by Kenneth Noland (Figure 7A) exhibits a high degree of co-circularity, which is a regularity that diminishes the 2nd-order entropy of the edge orientations (Geisler, 2008). The paintings by Piet Mondrian (Figure 7C) and Josef Albers (Figure 7D) are deviant because they contain horizontal and vertical lines exclusively (low edge orientation entropy) as well as large homogeneous areas (low complexity and self-similarity).

We speculate that the deviations in some *Bad Art* images might possibly arise because the artists lack training or talent in traditional art styles. As a consequence, the artists may fail to (or do not intend to) accomplish an image structure that is similar to that of prestigious traditional artworks. For abstract art, the deviating values in some images correspond to stylistic peculiarities (for example, see paintings in Figures 7A,C–F). We thus suggest that some (post-)modern artists transgressed the boundaries of traditional artworks, as defined in the present work by the four statistical image properties. This conclusion is in agreement with common art-historical and philosophical interpretations of (post-)modern art (for example, see Dickie, 1974; Danto, 1981), but it supports this notion with objective and quantitative evidence from a computer-based analysis of a large set of artworks.

However, not all stylistic specializations in abstract art result in deviations from the statistics of traditional artworks. Examples are the drip paintings by Jackson Pollock who created paintings with a highly complex, fractal (self-similar) structure (Taylor, 2002; Taylor et al., 2011). Although the resulting values for self-similarity and fractal dimension are higher than for traditional artworks, the other values for his painting remain within the range of traditional artworks. Importantly, the Mahalanobis distance to traditional artworks is relatively low (Figure 7H). Other artworks that, despite their distinctive abstract styles, have a spectrum of values similar to traditional artworks include paintings by Joan Miró (Figure 7J), Paul Klee (Figure 7K), Wassily Kandinsky (Figure 7L), Ernst Nay (Figure 7M), and others (not shown). We thus conclude that, as far as we can tell from the image properties measured in the present study, not all (post-)modern abstract artists abandoned the characteristic image structure of traditional art. On the contrary, the specific pattern of image properties in traditional art, which has prevailed for centuries and across different cultures, extends well into our modern times (Redies, 2014). Whether this pattern is necessary or sufficient to elicit the perception of visual beauty - or any particular aspects of visual aesthetics, such as liking, harmony, interest, aesthetic appeal, “good Gestalt” or “visual rightness”—remains to be studied by experimental means.

The present results are important because they provide, to our knowledge for the first time, an objective basis for a scientific comparison of traditional artworks that differ in their aesthetic claim, and for a study of the formal similarities and differences between the various types of traditional art and abstract art. Formal image analyses may complement and inform philosophical and art-historical analyses, which focus on contextual and cultural aspects of artworks (see Introduction). Formal image properties, such as 2nd-order edge entropy, are difficult to grasp and hard to describe in every-day terms. We propose that their investigation can provide access to an intuitive artistic knowledge that is based on perceptual mechanism and is largely inaccessible to cognitive introspection. To date, this hidden knowledge may have escaped the intellectual dissection of different art genres by contemporary art historians and art critics. Evidently, more research is needed to relate formal image statistics to art-historical knowledge, especially for (post-)modern and contemporary art.

## Author contributions

CR conceived and designed the experiment, assembled the novel image datasets, analyzed the data and wrote the manuscript. AB carried out the calculations and contributed to writing the manuscript.

### Conflict of interest statement

The authors declare that the research was conducted in the absence of any commercial or financial relationships that could be construed as a potential conflict of interest.

## Footnotes

^1^http://www.museumofbadart.org, accessed on February 10, 2017

^2^http://officialbadartmuseumofart.com, accessed on February 10, 2017

^3^http://officialbadartmuseumofart.com.

^4^http://www.tate.org.uk/search?q=abstract+art&type=artwork

^5^http://www.kunstsammlung.de/entdecken/sammlung/emuseum-sammlung.html
